# Supplementary material for: Cascade of fractional quantum Hall states in 2D system
Source: Natl Sci Rev. 2026 Feb 4;13(8):nwag079. doi: 10.1093/nsr/nwag079 (PMC13127151; doi:10.1093/nsr/nwag079)
Supplement: nwag079_Supplemental_File [file nwag079_supplemental_file.pdf]

# Supplemental Material for

## Cascade of fractional quantum Hall states in 2D systems

Zhimou Chen, Jiaojie Yan, Yuxuan Zhu, Zhe Cui, Loren N. Pfeiffer, Kenneth W. West, Kirk W. Baldwin, Adbhut Gupta, Yang Liu, Wei Zhu, Wenchen Luo, Ying-Hai Wu*, Shuai Yuan* and Xi Lin*

*Correspondence should be addressed to Y.-H.W. (yinghaiwu88@hust.edu.cn), S.Y. (shuaiy81@uw.edu) and X.L. (xilin@pku.edu.cn)

### 1. Illustrations of two possible $\nu$ = 6/5 states

The 6/5 FQH state could be an IQH state in the spin-down lowest LL plus a 1/5 FQH state in the spin-up lowest LL. The latter is the $\tilde{n}$ = 1 IQH state of CFs with four fluxes attached as shown in Fig. S1. If it is a two-component state, we first apply a particle-hole transformation to obtain a state at $2-\nu$ = 4/5. It is mapped to a $\tilde{n}$ = − 4/3 FQH state of CFs with two fluxes attached. Another particle-hole transformation relates 4/3 to the spin-singlet Jain state at 2/3 [1].

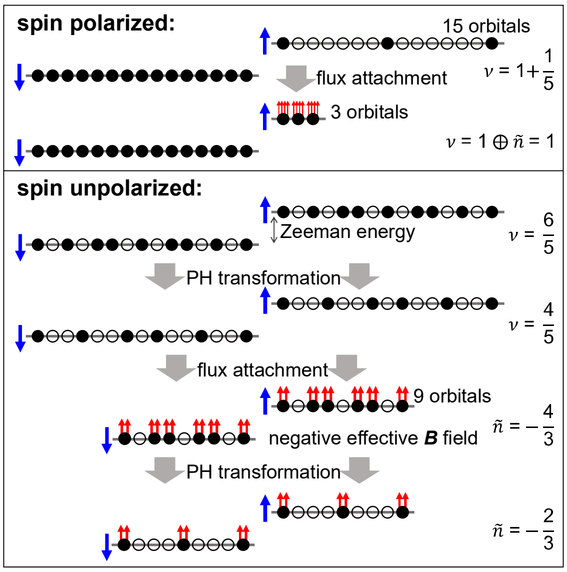


**Figure S1.** Schematics of two possible $\nu$ = 6/5 FQH states. In both cases, each LL has 15 orbitals and the number of electrons is 18. Blue arrows represent the spin direction of LLs, solid circles represent electrons, hollow circles represent vacant orbitals, and red arrows represent flux attached to electrons. Details about the constructions are given in the text.

### 2. Interpretation of experimental data using the hierarchy theory

The basic assumption of hierarchy theory is that the elementary charged excitations of one IQH or FQH state may form an incompressible state such that the whole system enters another FQH state[2]. Its starting point is usually taken as the $\nu$ = 1 IQH state or $\nu = 1/m$ Laughlin state. For example, a $\nu$ = 2/5 state is obtained when the quasiparticles of the 1/3 state form a bosonic $\nu$ = 1/2 Laughlin state. For spin-polarized electrons, this approach predicts that FQH states may be found at

$$\nu = \cfrac{1}{m + \cfrac{\alpha_1}{p_1 + \cfrac{\alpha_2}{p_2 + \dots}}} \tag{1}$$

with $m = 1, 3, 5, \cdots$ , $\alpha_n = 0, \pm 1$, and $p_n = 2, 4, 6, \cdots$ . The parameters in this formula are collected as $[m, \alpha_1, p_1, \cdots, \alpha_n, p_n]$.

We provide a sketch about how to explain the data in Fig. S2 using the hierarchy theory. For simplicity, only spin-polarized states are considered here. One principal sequence begins at $\nu = 1/3$ and traverses those at 2/5, 3/7, $\cdots$, $n/(2n+1)$. Another principal sequence begins at $\nu = 1$ and traverse those at 2/3, 3/5, $\cdots$, $n/(2n-1)$. While they terminate at $\nu = 1/2$, the hierarchy theory does not reveal the presence of composite fermion liquid. It is more interesting to study the states at $\nu = 4/11$, 4/13, and 5/13. For example, 4/11 can be obtained if the quasiparticles of the 1/3 state form a bosonic $\nu = 1/4$ Laughlin state. Its topological properties are expected to be the same as the FQH state of CFs in which the fractional part form a Laughlin state. However, previous works have found that the CFs may form an unconventional 1/3 state[3], and it is not obvious how to construct its equivalent in the hierarchy framework.

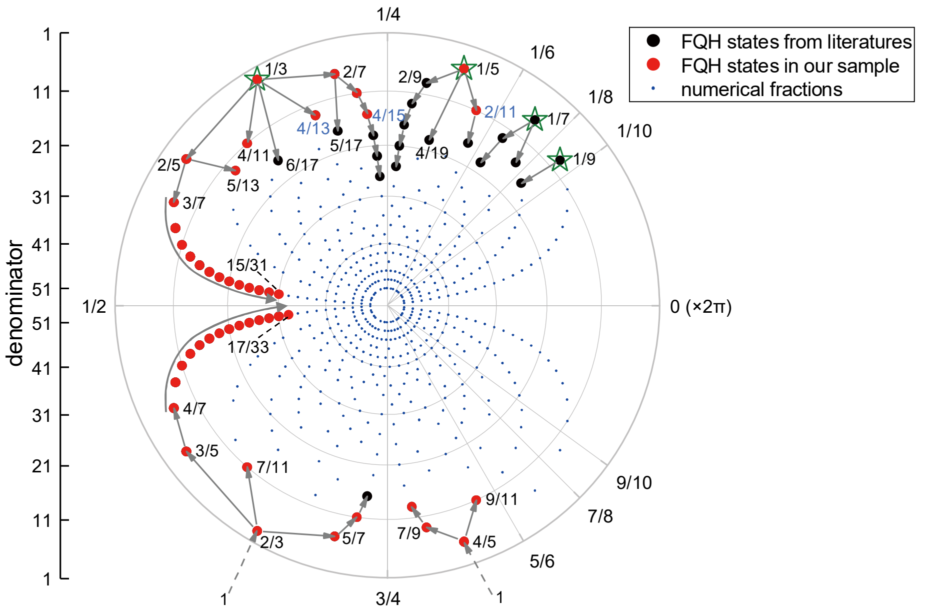


**Figure S2.** Interpretation of experimental data using the hierarchy theory. The same data points as in Fig. 2(a) are plotted[4–23]. Hollow green stars indicate the starting points of several hierarchies. Gray arrow lines indicate the path from a parent $[m, \alpha_1, p_1, \cdots, \alpha_{n\text{-}1}, p_{n\text{-}1}]$ to its daughter $[m, \alpha_1, p_1, \cdots, \alpha_{n\text{-}1}, p_{n\text{-}1}, \alpha_n, p_n]$. For the $\nu = 2/3$ and 4/5 states, $\nu = 1$ IQH state is their starting point and connected to them by dashed gray arrow line.

### 3. Numerical results and energy gaps at several filling factors

Exact diagonalization (ED) is a standard tool for studying FQH physics. It has played a decisive role in elucidating the nature of FQH states since the ground-breaking work of Laughlin. The basic idea of ED is straightforward. When a finite number of electrons are placed in a finite number of Landau orbitals, we construct all the Fock states corresponding to non-interacting Slater determinants. The many-body Hamiltonian is written in second quantized form and converted to a numeral matrix in the Fock states basis. If the system has symmetries, this matrix can be organized into a block diagonal form such that each block is treated separately. Using iterative sparse matrix eigensovler such as the Lanczos method, a few low-lying eigenvalues and eigenstates of the Hamiltonian

matrix can be obtained. One then proceeds to examine energy gaps, wave function overlaps, and entanglement properties etc. In general, ED can only be done for a small number of electrons because the Hilbert space dimension grows exponentially.

We have studied the $\nu$ = 13/11, 17/13, and 19/15 states using ED. The integer parts form IQH states whereas the fractional parts are assumed to be spin-polarized. It is much more difficult to include the spin degree of freedom because this would greatly enlarge the Hilbert space. To account for LL mixing effects, we employ both random phase approximation (RPA) and perturbation theory. The dielectric function in RPA is obtained by integrating out the neighboring LLs. In perturbative treatment of the contributions from higher LLs, two-body interactions are renormalized by zero-sound as well as Bardeen-Copper-Schrieffer diagrams, and three-body interactions arising from two screened vertices are considered[24]. The finite thickness of quantum wells is modeled by an infinite square well potential. Aided by these approximations, we obtain an effective single-LL description of the problem with a renormalized Coulomb interaction.

As shown in Fig. S3, the ground states for 8 electrons at $\nu$ = 13/11, 17/13 locate at $q \neq 0$ so the systems are compressible. This is consistent with the experimentally observed disappearance of the $R_{xx}$ dip at high in-plane magnetic fields. For $\nu$ = 19/15, preliminary numerical results suggest that an incompressible state may be stabilized. However, this claim should be viewed with caution because the system size is severely limited: only four momentum sectors are available. The true nature of the system can only be ascertained by more experimental investigations. At high in-plane magnetic fields, the $R_{xx}$ dip at $\nu$ = 19/15 disappears. This may be caused by a transition into a compressible state or because the energy gap is too small to be detected within our measurement capabilities.

We have performed Arrhenius fittings of the experimental data at different temperatures using the same method as in Ref.[25]. For tilt angle 30.9°, our fittings yielded energy gaps of approximately 32 mK at $\nu$ = 19/15 and 26 mK at $\nu$ = 17/13. These numbers are comparable to the base electron temperature of our rotatable sample stage (approximately 40 mK). This proximity in energy scales explains why we only observed weak dips in the $R_{xx}$ traces of Fig. 4(b). As for the $\nu$ = 13/11 state, the exceptionally large insulating background resistance disallows a valid Arrhenius fitting.

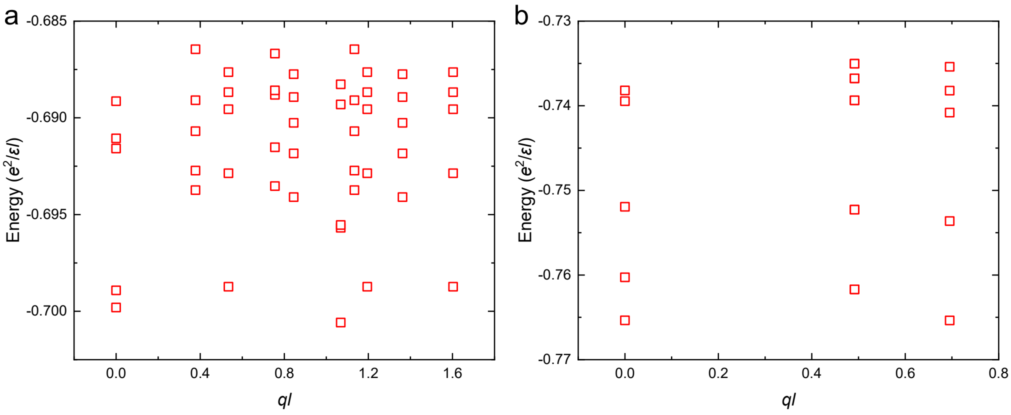


**Figure S3.** Numerical results for the (a) $\nu$ = 13/11 and (b) $\nu$ = 17/13 states with 8 spin-polarized electrons.

## 4. Filtering and electron temperature

The filters in the measurement leads play a crucial role in lowering the electron temperature of the sample. Our nuclear adiabatic demagnetization refrigerator is primarily used for quasi-DC measurements. Consequently, we have installed low-pass filters in the signal lines. We incorporated home-made silver-epoxy filters and RC filters in each measurement line. These two types of filters are connected in series with each other.

The filtering performance of our silver-epoxy filter and RC filter is consistent with the results reported in Ref.[26]. The attenuation is essentially better than -60 dB above 1 MHz and exceeds -90 dB above 1 GHz. Our silver epoxy filter is composed of a 0.1 mm diameter copper enameled wire encased in silver metal powder, and it operates primarily by utilizing the skin effect to filter high-frequency electromagnetic waves. The RC filter is a third-order configuration with respective resistor and capacitor values for each stage of (510 Ω, 4.7 nF), (820 Ω, 2.2 nF), and

(1500 Ω, 1.1 nF). The resistors and capacitors are mounted on an insulating substrate made of sapphire to ensure reasonable thermal contact with the cold plate. Due to the high resistor values, which could generate significant thermal noise at room temperature, the RC filter is mounted on the mixing chamber plate.

Based on the performance of the same sample in different refrigerators, we estimate that the electron temperature of the nuclear adiabatic demagnetization refrigerator is at least less than 12 mK. This is further corroborated by comparing the Hall resistance of several reentrant IQH states. As one can see from Fig. S4, the data collected in the nuclear demagnetization refrigerator have better quality, which again suggests that it reaches electron temperature lower than 12 mK (the value in our dilution refrigerator). On the other hand, it is quite likely that the conventional sample holder in our refrigerator is less efficient in cooling of the electron than the $^3$He immersion cell used in Ref.[23], which achieved an electron temperature of 4 mK.

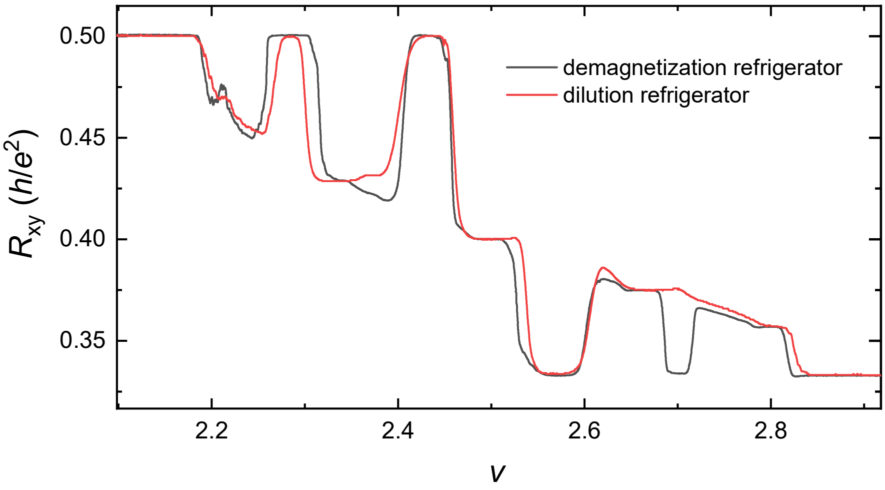


**Figure S4.** The Hall resistance of several reentrant IQH states in the second Landau level measured in the nuclear adiabatic demagnetization refrigerator and the dilution refrigerator.

**References**


1. Wu XG, Dev G, Jain JK. Mixed-spin incompressible states in the fractional quantum Hall effect. *Phys Rev Lett* 1993;**71**:153–6.

2. Haldane FDM. Fractional quantization of the Hall effect: a hierarchy of incompressible quantum fluid states. *Phys Rev Lett* 1983;**51**:605–8.

3. Mukherjee S, Mandal SS, Wu Y-H *et al.* Enigmatic 4/11 state: a prototype for unconventional fractional quantum Hall effect. *Phys Rev Lett* 2014;**112**:016801.

4. Chung YJ, Villegas Rosales KA, Baldwin KW *et al.* Ultra-high-quality two-dimensional electron systems. *Nat Mater* 2021;**20**:632–7.

5. Xia JS, Pan W, Vicente CL *et al.* Electron correlation in the second Landau level: a competition between many nearly degenerate quantum phases. *Phys Rev Lett* 2004;**93**:176809.

6. Willett RL, Stormer HL, Tsui DC *et al.* Termination of the series of fractional quantum Hall states at small filling factors. *Phys Rev B* 1988;**38**:7881–4.

7. Pan W, Stormer HL, Tsui DC *et al.* Transition from an electron solid to the sequence of fractional quantum Hall states at very low Landau level filling factor. *Phys Rev Lett* 2002;**88**:176802.

8. Wang C, Madathil PT, Singh SK *et al.* Developing fractional quantum Hall states at even-denominator fillings 1/6 and 1/8. *Phys Rev Lett* 2025;**134**:046502.

9. Huang H, Hussain W, Myers SA *et al.* Evidence for topological protection derived from six-flux composite fermions. *Nat Commun* 2024;**15**:1461.

10. Pan W, Stormer HL, Tsui DC *et al.* Fractional quantum Hall effect of composite fermions. *Phys Rev Lett* 2003;**90**:016801.

11. Pan W, Baldwin KW, West KW *et al.* Fractional quantum Hall effect at Landau level filling ν = 4/11. *Phys Rev B* 2015;**91**:041301.

12. Samkharadze N, Arnold I, Pfeiffer LN *et al.* Observation of incompressibility at ν = 4/11 and ν = 5/13. *Phys Rev B* 2015;**91**:081109.

13. Du RR, Tsui DC, Stormer HL *et al.* Fractional quantum Hall liquid to insulator transition in the vicinity of Landau level filling ν = 2/9. *Solid State Communications* 1996;**99**:755-7.

14. Pan W, Stormer HL, Tsui DC *et al.* Some fractions are more special than others: news from the fractional quantum Hall zone. *International Journal of Modern Physics B* 2002;**16**:2940–5.

15. Gervais G, Engel LW, Stormer HL *et al.* Competition between a fractional quantum Hall liquid and bubble and Wigner crystal phases in the third Landau level. *Phys Rev Lett* 2004;**93**:266804.

16. Choi HC, Kang W, Das Sarma S *et al.* Activation gaps of fractional quantum Hall effect in the second Landau level. *Phys Rev B* 2008;**77**:081301.

17. Pan W, Xia JS, Stormer HL *et al.* Experimental studies of the fractional quantum Hall effect in the first excited Landau level. *Phys Rev B* 2008;**77**:075307.

18. Zhang C, Huan C, Xia JS *et al.* Spin polarization of the ν = 12/5 fractional quantum Hall state. *Phys Rev B* 2012;**85**:241302.

19. Kleinbaum E, Kumar A, Pfeiffer LN *et al.* Gap reversal at filling factors 3 + 1/3 and 3 + 1/5: towards novel topological order in the fractional quantum Hall regime. *Phys Rev Lett* 2015;**114**:076801.

20. Shingla V, Kleinbaum E, Kumar A *et al.* Finite-temperature behavior in the second Landau level of the two-dimensional electron gas. *Phys Rev B* 2018;**97**:241105.

21. Chung YJ, Graf D, Engel LW *et al.* Correlated states of 2D electrons near the Landau level filling ν = 1/7. *Phys Rev Lett* 2022;**128**:026802.

22. Kumar A, Csáthy GA, Manfra MJ *et al.* Nonconventional odd-denominator fractional quantum Hall states in the second Landau level. *Phys Rev Lett* 2010;**105**:246808.

23. Samkharadze N, Kumar A, Manfra MJ *et al.* Integrated electronic transport and thermometry at milliKelvin temperatures and in strong magnetic fields. *Review of Scientific Instruments* 2011;**82**:053902.

24. Luo W, Abdulwahab M, Liu X *et al.* 5/2 fractional quantum Hall state in GaAs with Landau level mixing. *Phys Rev B* 2024;**110**:085428.

25. Wang C, Gupta A, Chung YJ *et al.* Highly anisotropic even-denominator fractional quantum Hall state in an orbitally coupled half-filled Landau level. *Phys Rev Lett* 2023;**131**:056302.

26. Wang P, Huang K, Sun J *et al.* Piezo-driven sample rotation system with ultra-low electron temperature. *Review of Scientific Instruments* 2019;**90**:023905.
